# Urinary Titin Is Increased in Patients After Cardiac Surgery

**DOI:** 10.3389/fcvm.2019.00007

**Published:** 2019-02-08

**Authors:** Jun Tanihata, Naritomo Nishioka, Takahiro Inoue, Ko Bando, Susumu Minamisawa

**Affiliations:** ^1^Department of Cell Physiology, The Jikei University School of Medicine, Tokyo, Japan; ^2^Department of Cardiac Surgery, The Jikei University School of Medicine, Tokyo, Japan

**Keywords:** biomarker, urine, myocardial injury, cardiac surgery, sarcomeric protein, titin

## Abstract

**Background:** Few non-invasive biomarkers have been used to detect myocardial injury in patients with heart diseases. Recently, the N-terminal fragment (N-titin) of titin, a giant sarcomeric protein, which is involved in muscular passive tension and viscoelasticity, has been reported to detect muscle damage in patients with cardiomyopathy as well as in patients with skeletal muscle dystrophy and in healthy volunteers with endurance exercise. In the present study, we evaluated whether urinary N-titin is changed during a perioperative period and whether its increase reflects myocardial damage.

**Materials and Methods:** In 18 patients who underwent cardiac surgery, blood and urine samples were obtained before and after surgery. We measured the urinary levels of N-titin with a highly sensitive ELISA system.

**Results:** Urinary N-titin to creatinine (N-titin/Cr) was significantly increased in all patients postoperatively (43.3 ± 39.5 pmol/mg/dL on the day of operation) and remained significantly high for at least 4 days postoperatively. Urinary N-titin/Cr was positively correlated with serum cardiac troponin T (*r* = 0.36, *p* = 0.0006, *n* = 90) but not creatine kinase-MB (CK-MB). We also found that urinary N-titin/Cr in patients after a coronary artery bypass grafting operation was higher by day 2 postoperatively than in patients following open cardiac surgeries.

**Conclusion:** The cleaved N-titin was significantly increased in urine after cardiac surgery. Urinary N-titin may be useful for detecting the risk of latent postoperative cardiac damage.

## Introduction

Biomarkers for cardiac damage such as high-sensitive troponin T have been widely used to manage patients with heart diseases including perioperative ischemia (1–4). These biomarkers are mostly measured using serum samples and there are few non-invasive biomarkers for detecting myocardial injury. Zhou et al. recently demonstrated that microRNA-1 (miR-1), a cardio-specific/enriched microRNA, is increased in urine after open-heart surgeries with cardiopulmonary bypass, indicating that urinary miR-1 can be a novel non-invasive biomarker for myocardial injury (5). More recently, Yoshihisa et al. reported that urinary N-terminal fragment (N-titin) of titin, a giant sarcomeric protein that is involved in muscular passive tension and viscoelasticity, could be a biomarker for predicting high risk dilated cardiomyopathy patients (6) by using the enzyme-linked immunosorbent assay (ELISA) system (7). In accordance with this finding, plasma titin is closely associated with the onset of acute myocardial infarction in patients with type 2 diabetes mellitus (8).

Biomarkers are also useful for patients after cardiac surgery. This type of surgery may inadvertently cause perioperative myocardial injury, which can substantially increase the risk of postoperative morbidity and mortality. Even after successful operations, there is the potential risk of latent myocardial injury because the molecular markers of cardiac damage such as creatine kinase-MB (CK-MB) and cardiac troponin isoforms are known to transiently increase postoperatively (2–4). However, whether urinary N-titin is changed during the perioperative period and whether its increase reflects myocardial damage has yet to be tested. In the present study, we hypothesized that urinary N-titin could be a good indicator for detecting myocardial injury due to cardiac surgery and could also be useful for postoperative patient care. To evaluate our hypothesis, urinary N-titin was measured in patients who underwent cardiac surgery and was compared with other parameters including serum biomarkers such as troponin T and CK-MB.

## Materials and Methods

### Patients

After obtaining written informed consents, 18 adult patients (15 male, 3 female; 68 ± 12.7 years old) who had undergone cardiac surgery from 2017/11/27 to 2018/2/6 participated in this study. The Ethics Committee of The Jikei University School of Medicine approved the present study (Protocol Number: 29-095[8711]). Patients with recent myocardial infarction; unstable angina; preoperative congestive heart failure (CHF), unstable arrhythmias; diabetes mellitus, or hepatic, renal, or neurological dysfunction were excluded from this study. There were no operation-related deaths or serious complications in this patient cohort. Procedures included 5 open cardiac surgeries, 10 on-pump beating coronary artery bypass graft (CABG) surgeries, and 3 off-pump CABG surgeries (Supplemental Table 1).

### Blood and Urine Sample Collection and Measurement of N-titin

Blood and urine samples were obtained at pre-surgery (pre), and postoperatively from days 0 to 3. In addition, we collected urine samples at day 4 postoperatively. Serum and urine samples were stored at −20°C until analysis. We measured the urinary levels of N-titin with a highly sensitive ELISA system (#27900 Titin N-Fragment Assay Kit; Immuno-Biological Laboratories Co. Ltd., Japan) according to the manufacturer's instructions. Detailed data of this system were previously reported (7). To avoid the effects of urinary filtration, the value of N-titin concentration was corrected by the value of creatinine, and is shown by the following creatinine ratio: (N-titin/Cr; pmol/mg/dl) = N-titin (nmol/L)/creatinine (mg/dl) × 100 (7). Serum cardiac troponin T levels, creatine kinase-MB (CK-MB) and total creatine kinase (CK) were measured using the electro chemiluminescence immunoassay (SRL, Inc) and ultraviolet absorption spectrophotometry (SRL, Inc), respectively. We calculated the value of CK-MM from the fraction of CK-MB to total CK.

### Statistical Analysis

All data are presented as mean ± SD. For relative gene expression, the mean value of the control group was defined as 1. Differences in N-titin levels were analyzed using one-way ANOVA with Bonferroni's multiple comparisons *post hoc* test. Linear regression analysis was used to determine the relationship between urinary N-titin and serum cardiac troponin T or CK-MB. A *p* < 0.05 was considered significant.

## Results

### N-titin Was Significantly Increased After Cardiac Surgery

There were no operation-related deaths or serious complications in this patient cohort. Procedures included 5 open cardiac surgeries, 10 on-pump beating coronary artery bypass graft (CABG) surgeries, and 3 off-pump CABG surgeries (Supplemental Table 1).

**Figure 1:**
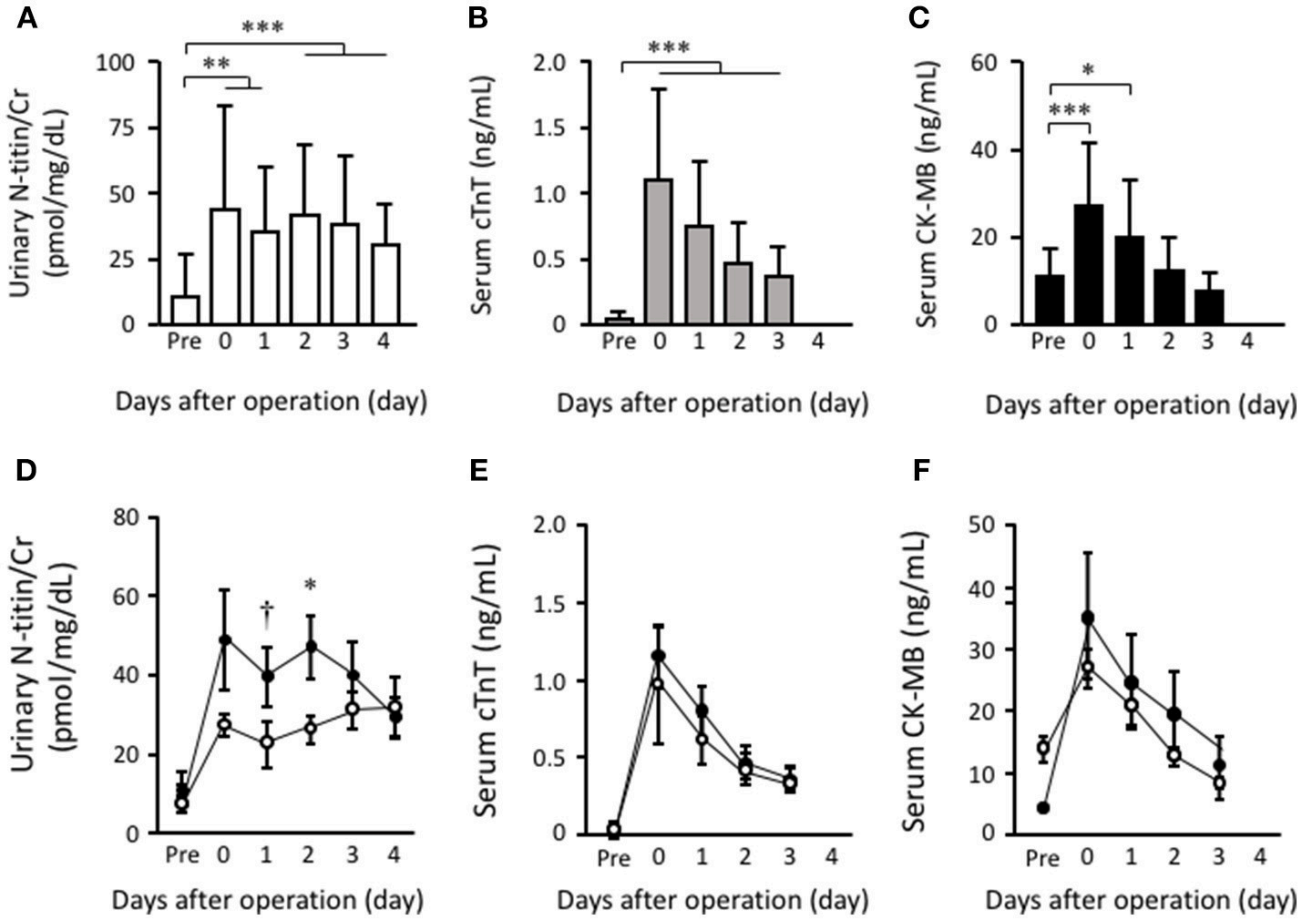
The effects of the surgical procedure on urinary N-titin/Cr, plasma cardiac troponin T and plasma CK-MB. Time course changes in values of urinary N-titin/Cr values **(A)**, plasma cardiac troponin T **(B)** and plasma CK-MB **(C)** pre- and postoperatively. Time course changes in values of urinary N-titin/Cr values **(D)**, plasma cardiac troponin T **(E)**, and plasma CK-MB **(F)** by CABG (black circle) and open cardiac surgery (white circle) pre- and postoperatively. Data are presented as means ± SD. ^†^*p* < 0.1, ^*^p < 0.05, ^**^P < 0.01, and ^***^P < 0.001 by one-way ANOVA with Bonferroni's multiple comparisons *post hoc* test.

The preoperative ratio of urinary N-titin to creatinine (N-titin/Cr) was 10.0 ± 15.9 pmol/mg/dL, and it was significantly increased in all patients postoperatively (43.3 ± 39.5 pmol/mg/dL on the day of operation) (Figure 1A). Preoperative cardiac troponin T was 0.033 ± 0.063 ng/mL, and it was significantly increased in all patients postoperatively (1.100 ± 0.700 ng/mL on the day of operation) (Figure 1B). Preoperative serum CK-MB (*n* = 10) was 11.2 ± 6.4 U/L, and it was also significantly increased in all 17 patients postoperatively (27.1 ± 14.8 U/L on the day of operation) (Figure 1C).

Urinary N-titin/Cr (37.9 ± 26.1 pmol/mg/dL) and serum cardiac troponin T (0.361 ± 0.233 ng/mL) were still significantly high at 3 days postoperatively when compared to the preoperative value, whereas serum CK-MB rapidly decreased to the preoperative level by 3 days postoperatively.

We then investigated the effect of the surgical procedure on urinary N-titin. In CABG patients, there were 10 patients who underwent the on-pump beating procedure and 3 who underwent the off-pump procedure. There was no significant difference in urinary N-titin/Cr between on-pump and off-pump CABG patients. Therefore, we next compared all CABG patients with patients after open cardiac surgery. Interestingly, urinary N-titin/Cr at postoperatively day 2 was higher in CABG patients than in open cardiac surgery patients (Figure 1D). The difference was statistically significant at postoperative days 1 and 2. Although cardiac troponin T and CK-MB at postoperative days 0 and 1 was slightly higher in CABG patients than in open cardiac surgery patients, there was no statistical significance (Figures 1E,F). On the other hand, serum CK-MM values increased on the 2 and 3 days postoperatively in CABG and open cardiac surgery patients, although it was not increased by 1 days after cardiac surgery (Supplemental Figure 1). Regarding the perioperative characteristics, we found that there was no significant correlation between the peak value of urinary N- titin/Cr and other parameters including operation time (*r* = 0.02, *p* = 0.940, *n* = 14), cardiopulmonary bypass time (*r* = −0.15, *p* = 0.800, *n* = 5), duration of intensive care stay (*r* = −0.09, *p* = 0.733, *n* = 17), postoperative left ventricular ejection fraction (*r* = −0.37, *p* = 0.177, *n* = 15), and age (*r* = 0.35, *p* = 0.156, *n* = 18) (Supplemental Figure 2).

### The Correlation of Urinary N-titin and Serum Biomarkers

**Figure 2:**
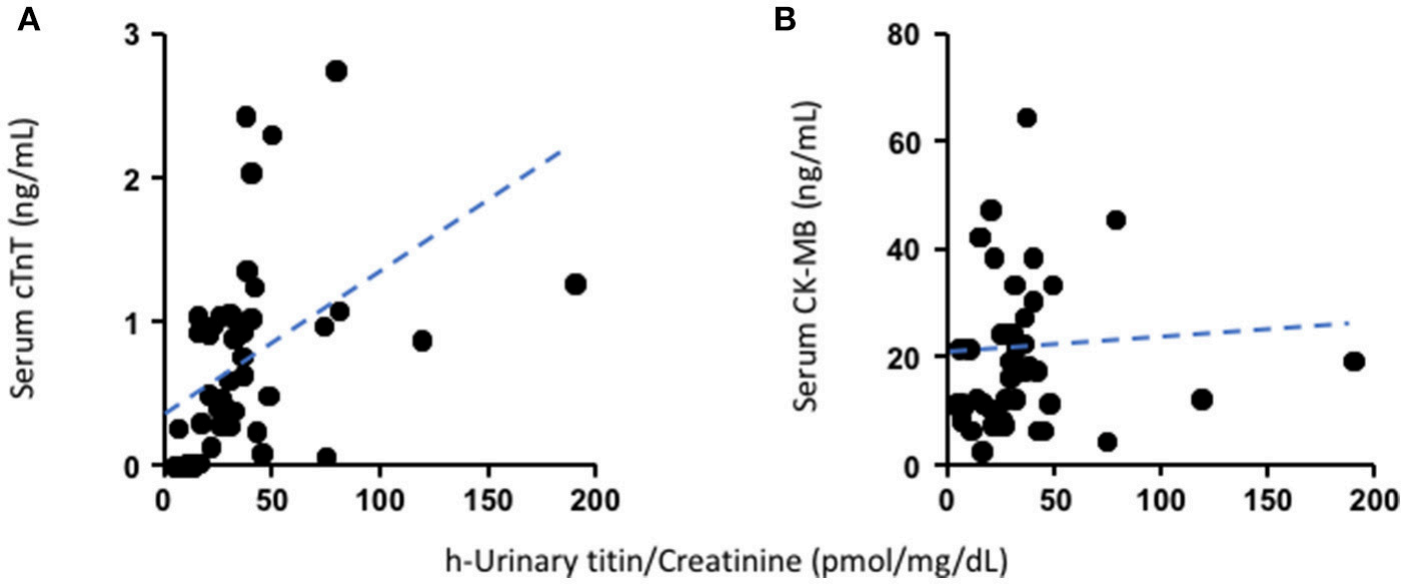
The relationship between urinary N-titin/Cr and serum cardiac troponin T or CK-MB levels preoperatively and at postoperatively days 0 and 1. **(A)** The relationship between urinary N-titin/Cr and serum cardiac troponin T in patients who underwent cardiac surgery. A positive correlation was demonstrated between the two variables (*r* = 0.47, *p* = 0.0004, *n* = 54). **(B)** The relationship between urinary N-titin/Cr and serum CK-MB in patients who underwent cardiac surgery. No correlation was demonstrated between the two variables (*r* = 0.06, *p* = 0.72, *n* = 44).

We analyzed the association correlations of urinary N-titin/Cr with serum cardiac troponin T or CK-MB with Pearson's correlation coefficient (r) in the perioperative period at postoperative day 1. There were positive correlations between urinary N-titin/Cr and serum cardiac troponin T (*r* = 0.47, *p* = 0.0004, *n* = 54) (Figure 2A), but not between N-titin/Cr and CK-MB (*r* = 0.06 *p* = 0.717, *n* = 44) (Figure 2B). We also analyzed the association correlations of these parameters at postoperative days 2 and 3. On the contrary, there were no correlations between urinary N-titin/Cr and serum cardiac troponin T (*r* = 0.21, *p* = 0.217, *n* = 36) (Supplemental Figure 3A) or CK-MB (*r* = −0.14, *p* = 0.521, *n* = 25) (Supplemental Figure 3B). If we analyzed all samples including samples at postoperative days 2 and 3, there were lower positive correlations between urinary N-titin/Cr and serum cardiac troponin T (*r* = 0.36, *p* = 0.0006, *n* = 90) (Supplemental Figure 3C), whereas there was no correlation between urinary N-titin/Cr and CK-MB (*r* = −0.03, *p* = 0.841, *n* = 69) (Supplemental Figure 3D).

## Discussion

We found for the first time that urinary N-titin/Cr significantly increased after cardiac surgery and maintained high values at least 4 days postoperatively. Furthermore, urinary N-titin/Cr closely correlated with serum cardiac troponin T, which is a sensitive biomarker for myocardial damage in an acute phase of cardiac operation. Titin is a giant sarcomeric protein that is involved in myocardial passive tension and viscoelasticity. The giant titin protein is known to be cleaved by calpain and matrix-metalloproteinase-2 when protease activity is increased in pathological conditions such as oxidative stress (9). Recent studies have demonstrated that the cleaved titin fragment can be detected in striated muscles in urine (6, 7, 10, 11), indicating that the level of titin fragments in urine is associated with muscle damage. Previous studies have demonstrated that serum troponin isoforms were increased after cardiac surgery even in successful operation cases (2–4), which is consistent with the present study. We found strong correlations between urinary N-titin/Cr and serum cardiac troponin T immediately after cardiac surgery, indicating that, at least by postoperative day 1, the main origin of urinary N-titin was the heart. These results suggest that subtle myocardial damage due to cardiac surgery might be hidden. The present result indicated that an increase in N-titin in urine reflected subtle myocardial damage even though we did not observe any significant functional change in our patients. The advantages of the ELISA kit we used in this experiment are that it has been commercially available for human samples and it takes <3 h to obtain the results.

In the present study, we found that urinary N-titin/Cr did not differ between on-pump and off-pump CABG patients. A considerable number of studies have demonstrated that off-pump CABG provides better myocardial protection than on-pump CABG does (12–14). The reason for this discrepancy might be due to the small number of patients in our study. In addition, it should be noted that we used an on-pump beating procedure which is different from the conventional on-pump CABG in which the heart is completely arrested. Furthermore, we found that urinary N-titin/Cr in CABG patients was higher by postoperative day 2 than in postoperative open cardiac surgery patients. Because the other non-cardiac factors, such as surgical incision, did not differ between the groups, the result suggested that latent myocardial damage might be greater in patients after CABG than in patients after open cardiac surgery. In our institute, cardioplegia is not used for CABG operations, because all CABG surgery is performed under beating conditions with or without cardiopulmonary bypass circuits. In addition, cardiac ischemia might occur because a certain volume of coronary blood flow would be stolen during the suturing of the coronary artery. These factors might cause latent myocardial damage more frequently in CABG patients than in open cardiac surgery patients.

## Limitations of the Present Study

Several limitations of the present study must be discussed regarding the use of urinary titin as a biomarker of myocardial injury after cardiac surgery. First, the sample size in this study may be too small for a heterogenous patient cohort to establish the clinical significance of urinary titin as a biomarker. This is primarily due to the short period of the study. In addition, we strictly excluded patients who had recent myocardial infarction, unstable angina, preoperative CHF, unstable arrhythmias, diabetes mellitus, or hepatic, renal, or neurological dysfunction. Nevertheless, we believe that our findings are valuable because our primary purpose was to demonstrate for the first time that urinary titin has potential as an alternative, non-invasive biomarker for myocardial injury after cardiovascular surgery. Further investigation with a larger number of cases is absolutely necessary to clarify the clinical significance of urinary titin as a useful biomarker after cardiac surgery. The second limitation is that we could not detect serum titin in our patients, despite Rahim et al. reporting that the 35 kDa titin fragment was detected in serum (8). On the other hand, from the ELISA kit we used in this experiment, the size of the detected titin fragment was 26 kDa in urine. According to the manufacturer's protocol, the ELISA kit cannot detect the titin fragment in serum due to unknown reasons. We assume that some factors in the serum may inhibit the immunoreaction of the ELISA kit.

The third limitation is that the overall correlation between the urinary titin concentration and a current standard myocardial injury biomarker such as serum cardiac troponin T is relatively low because there was no significant correlation between urinary N-titin/Cr and serum cardiac troponin T at postoperative days 2 and 3. Urinary N-titin/Cr remained high even 4 days postoperatively, whereas serum cardiac troponin T rapidly returned to its preoperative level. We assume that this discrepancy arose from the skeletal muscle damage such as that caused by incision and may possibly affect the increase in urinary titin/Cr in a relatively late phase after cardiac surgery. Another possibility is that the serum concentrations of N-titin might be maintained for a longer period than those of cardiac troponin T such as myosin light chain 1. It has been shown that serum myosin light chain 1 maintains high levels for at least 1 week after myocardial infarction (15, 16). However, we cannot prove these assumptions because the ELISA system developed by Maruyama et al. (7) can detect both cardiac and skeletal N-titin. Therefore, to test the latter possibility, we tried to measure serum N-titin using the same ELISA system. However, as mentioned above, we have so far failed to detect a measurable level of N-titin in serum samples. To overcome these limitations, a new assay system that allows us to detect cardiac isoform-specific titin fragments in both serum and urine is needed to clarify the source (cardiac or skeletal). Lastly, the practicability of using a urinary biomarker may be limited for the perioperative course of cardiac operations because urinary titin measurements cannot be applied to patients with renal dysfunction after prolonged cardiomyopathy bypass or to those with renal failure.

## Conclusion

We found that urinary N-titin/Cr significantly increased after cardiac surgery and maintained high values at least 4 days postoperatively. Urinary N-titin/Cr may be useful for detecting the risk of substantial postoperative cardiac damage.

## Author Contributions

SM conceived of the study. TI, KB, and SM designed the experiments. JT, NN, and SM wrote the manuscript. JT, NN, and TI performed the experiments. All authors discussed the results and implications and commented on the manuscript at all stages of its development.

### Conflict of Interest Statement

The authors declare that the research was conducted in the absence of any commercial or financial relationships that could be construed as a potential conflict of interest.

## Supplementary Material

Supplemental Figure 1 The effects of the surgical procedure on plasma CK-MM. Time course changes in values of plasma CK-MM by CABG (black circle) and open cardiac surgery (white circle) pre- and postoperatively. Data are presented as means ± SD.

Supplemental Figure 2 The relationship between the peak values of urinary N-titin/Cr and cardiopulmonary bypass time, cross-clamp time, duration of intensive care stay, postoperative left ventricular ejection fraction (EF) and age. **(A)** The relationship between the peak values of urinary N-titin/Cr and cardiopulmonary bypass time in patients who underwent cardiac surgery. No correlation was demonstrated between the two variables (*r* = 0.02, *p* = 0.940, *n* = 14). **(B)** The relationship between the peak values of urinary N-titin/Cr and cross-clamp time in patients who underwent cardiac surgery. No correlation was demonstrated between the two variables (*r* = −0.154, *p* = 0.804, *n* = 5). **(C)** The relationship between the peak values of urinary N-titin/Cr and duration of intensive care stay in patients who underwent cardiac surgery. No correlation was demonstrated between the two variables (*r* = −0.09, *p* = 0.733, *n* = 17). **(D)** The relationship between the peak values of urinary N-titin/Cr and left ventricular ejection fraction following cardiac surgery. No correlation was demonstrated between the two variables (*r* = −0.37, *p* = 0.178, *n* = 15). **(E)** The relationship between the peak values of urinary N-titin/Cr and patient age. No correlation was demonstrated between the two variables (*r* = 0.35, *p* = 0.156, *n* = 18).

Supplemental Figure 3 The relationship between urinary N-titin/Cr and serum cardiac troponin T or CK-MB levels on postoperative days 2 and 3 on all samples. **(A)** The relationship between urinary N-titin/Cr and serum cardiac troponin T in patients with cardiac surgery on postoperative days 2 and 3. No correlation was demonstrated between the two variables (*r* = 0.21, *p* = 0.217 *n* = 36). **(B)** The relationship between urinary N-titin/Cr and serum CK-MB in patients who underwent cardiac surgery on postoperative days 2 and 3. No correlation was demonstrated between the two variables (*r* = −0.14, *p* = 0.521, *n* = 25). **(C)** The relationship between urinary N-titin/Cr and serum cardiac troponin T in patients with cardiac surgery from all samples. A positive correlation was demonstrated between the two variables (*r* = 0.36, *p* = 0.0006, *n* = 90). **(D)** The relationship between urinary N-titin/Cr and serum CK-MB in patients who underwent cardiac surgery from all samples. No correlation was demonstrated between the two variables (*r* = −0.03, *p* = 0.841, *n* = 69).

Supplement Table 1 Patient list used for this study.

## Abbreviations

PVE: prosthetic valve endocarditis
AAE: annuloaortic ectasia
AR: aortic regurgitation
IE: infectious endocarditis
MR: mitral regurgitation
TR: tricuspid regurgitation
AVR: aortic valve replacement
MVP: mitral valve plasty
TAP: tricuspid annuloplasty
AVP: aortic valvuloplasty
VAP: variant angina pectoris
EAP: effort angina pectoris
AP: angina pectoris
ACS: acute coronary syndrome
MI: myocardial infarction
SMI: silent myocardial infarction
OMI: old myocardial infarction
CABG: coronary artery bypass grafting.
