# Supplementary figures and images for: Urinary Titin Is Increased in Patients After Cardiac Surgery
Source: Front Cardiovasc Med. 2019 Feb 8;6:7. doi: 10.3389/fcvm.2019.00007 (PMC6375839; doi:10.3389/fcvm.2019.00007)

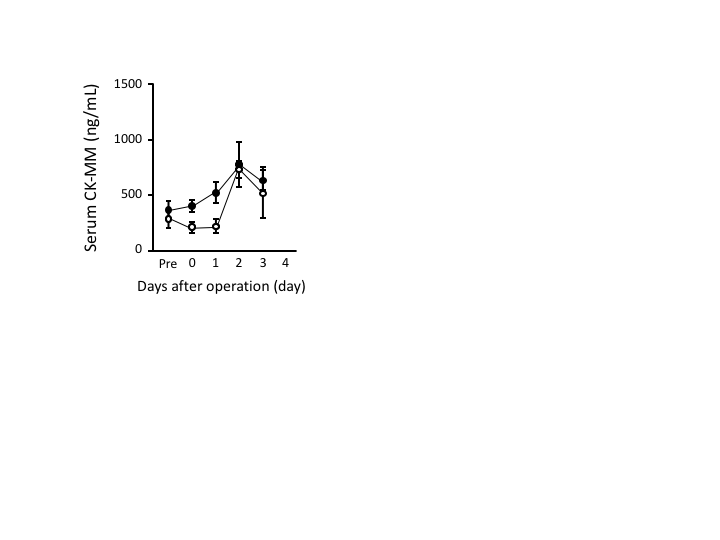

Supplement: Supplemental Figure 1 — The effects of the surgical procedure on plasma CK-MM. Time course changes in values of plasma CK-MM by CABG (black circle) and open cardiac surgery (white circle) pre- and postoperatively. Data are presented as means ± SD. [file Image_1.TIFF]
