# Supplementary material for: Urinary Titin Is Increased in Patients After Cardiac Surgery
Source: Front Cardiovasc Med. 2019 Feb 8;6:7. doi: 10.3389/fcvm.2019.00007 (PMC6375839; doi:10.3389/fcvm.2019.00007)
Supplement: Supplemental Figure 2 — The relationship between the peak values of urinary N-titin/Cr and cardiopulmonary bypass time, cross-clamp time, duration of intensive care stay, postoperative left ventricular ejection fraction (EF) and age. (A) The relationship between the peak values of urinary N-titin/Cr and cardiopulmonary bypass time in patients who underwent cardiac surgery. No correlation was demonstrated between the two variables (r = 0.02, p = 0.940, n = 14). (B) The relationship between the peak values of urinary N-titin/Cr and cross-clamp time in patients who underwent cardiac surgery. No correlation was demonstrated between the two variables (r = −0.154, p = 0.804, n = 5). (C) The relationship between the peak values of urinary N-titin/Cr and duration of intensive care stay in patients who underwent cardiac surgery. No correlation was demonstrated between the two variables (r = −0.09, p = 0.733, n = 17). (D) The relationship between the peak values of urinary N-titin/Cr and left ventricular ejection fraction following cardiac surgery. No correlation was demonstrated between the two variables (r = −0.37, p = 0.178, n = 15). (E) The relationship between the peak values of urinary N-titin/Cr and patient age. No correlation was demonstrated between the two variables (r = 0.35, p = 0.156, n = 18). [file Presentation_2.pptx]

## Slide 1
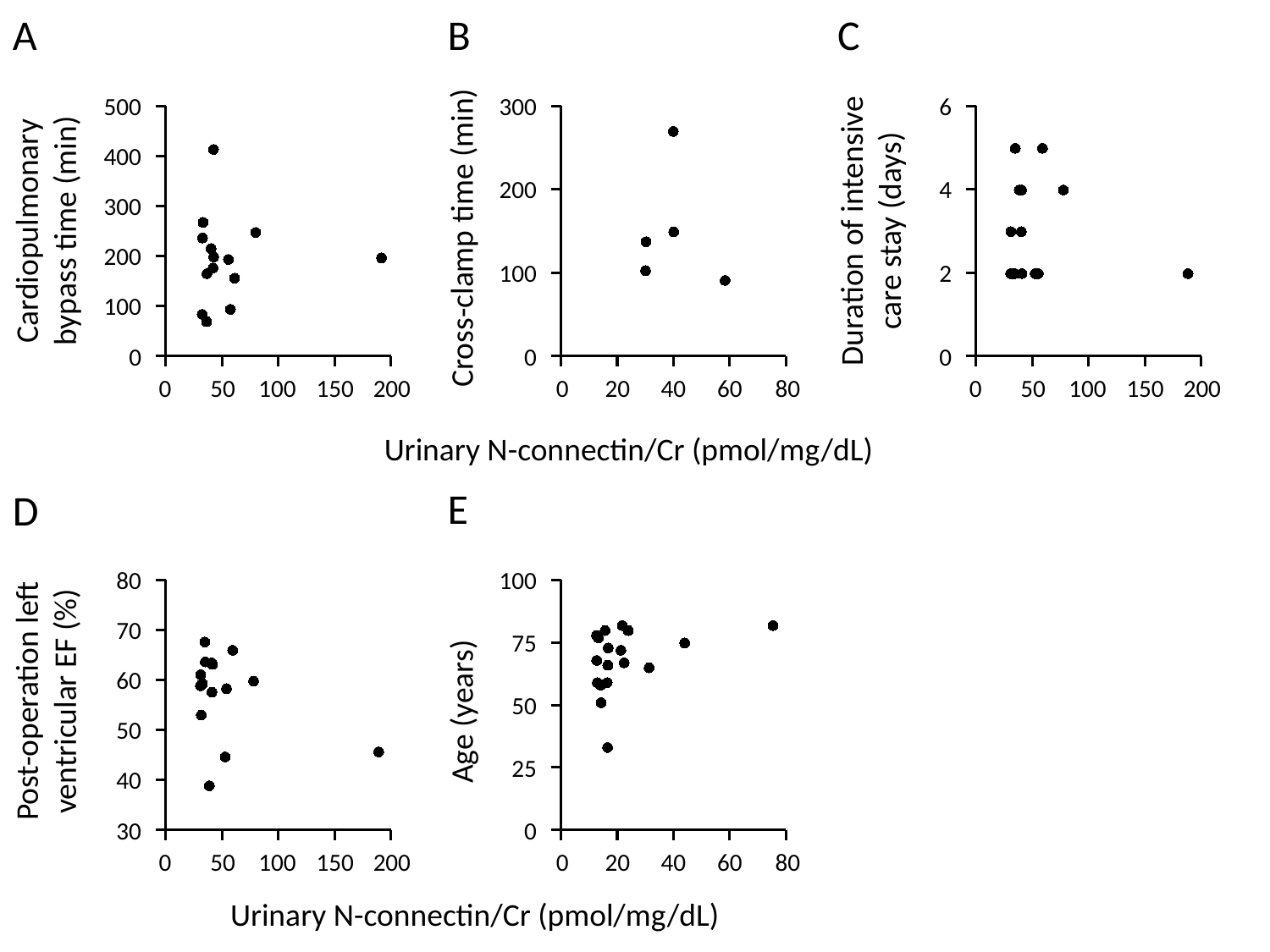

A
B
C
500
300
6
400
200
4
300
Cardiopulmonary bypass time (min)
Duration of intensive care stay (days)
Cross-clamp time (min)
200
100
2
100
0
0
0
0
50
100
150
200
0
20
40
60
80
0
50
100
150
200
Urinary N-connectin/Cr (pmol/mg/dL)
E
D
80
100
70
75
Post-operation left ventricular EF (%)
60
50
Age (years)
50
25
40
30
0
0
50
100
150
200
0
20
40
60
80
Urinary N-connectin/Cr (pmol/mg/dL)
