# Supplementary material for: Urinary Titin Is Increased in Patients After Cardiac Surgery
Source: Front Cardiovasc Med. 2019 Feb 8;6:7. doi: 10.3389/fcvm.2019.00007 (PMC6375839; doi:10.3389/fcvm.2019.00007)
Supplement: Supplemental Figure 3 — The relationship between urinary N-titin/Cr and serum cardiac troponin T or CK-MB levels on postoperative days 2 and 3 on all samples. (A) The relationship between urinary N-titin/Cr and serum cardiac troponin T in patients with cardiac surgery on postoperative days 2 and 3. No correlation was demonstrated between the two variables (r = 0.21, p = 0.217 n = 36). (B) The relationship between urinary N-titin/Cr and serum CK-MB in patients who underwent cardiac surgery on postoperative days 2 and 3. No correlation was demonstrated between the two variables (r = −0.14, p = 0.521, n = 25). (C) The relationship between urinary N-titin/Cr and serum cardiac troponin T in patients with cardiac surgery from all samples. A positive correlation was demonstrated between the two variables (r = 0.36, p = 0.0006, n = 90). (D) The relationship between urinary N-titin/Cr and serum CK-MB in patients who underwent cardiac surgery from all samples. No correlation was demonstrated between the two variables (r = −0.03, p = 0.841, n = 69). [file Presentation_3.pptx]

## Slide 1
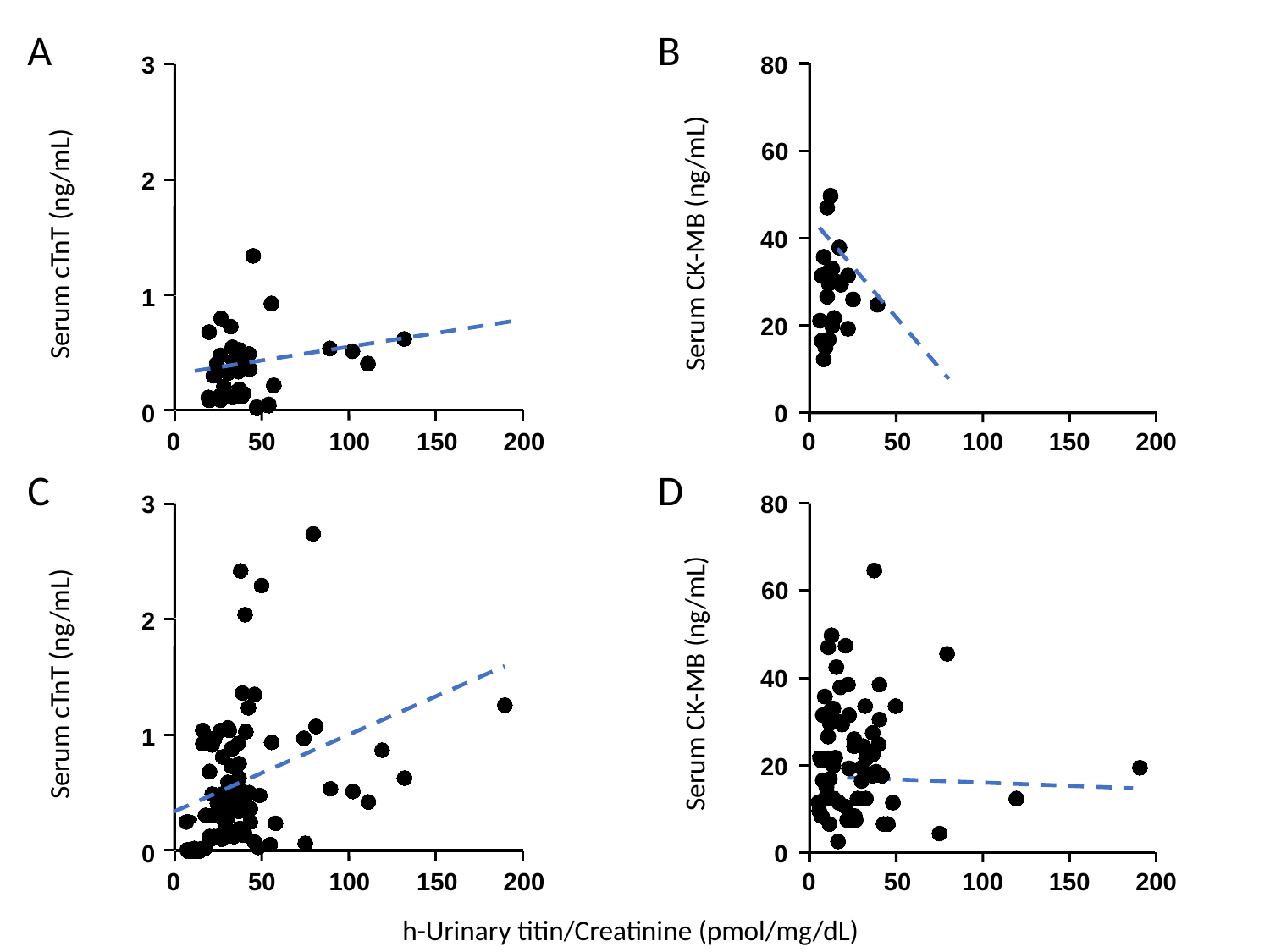

A
B
3
80
60
2
Serum CK-MB (ng/mL)
Serum cTnT (ng/mL)
40
1
20
0
0
0
50
100
150
200
0
50
100
150
200
C
D
3
80
60
2
Serum CK-MB (ng/mL)
Serum cTnT (ng/mL)
40
1
20
0
0
0
50
100
150
200
0
50
100
150
200
h-Urinary titin/Creatinine (pmol/mg/dL)
